# Obstructive sleep apnoea syndrome (OSAS) as a risk factor for secondary osteoporosis in children

**DOI:** 10.1038/s41598-021-82605-6

**Published:** 2021-02-04

**Authors:** Nur Syazwin Sies, Azriyanti Anuar Zaini, Jessie Anne de Bruyne, Muhammad Yazid Jalaludin, Anna Marie Nathan, Ng Yit Han, Surendran Thavagnanam

**Affiliations:** 1 Department of Paediatrics, University of Malaya, Kuala Lumpur, Malaysia; 2 University Malaya Paediatric and Child Health Research Group, University of Malaya, Kuala Lumpur, Malaysia; 3 Public Health Unit, Department of Social and Preventive Medicine, Faculty of Medicine, University of Malaya, Kuala Lumpur, Malaysia; 4 Department of Paediatrics, Royal London Hospital, London, UK

**Keywords:** Medical research, Paediatric research

## Abstract

Repetitive hypoxia seen in obstructive sleep apnoea syndrome (OSAS) may affect bone metabolism increasing the risk for secondary osteoporosis. This study investigates the association between OSAS in children and secondary osteoporosis. This cross-sectional study included 150 children aged 10–17 years: 86 with OSAS and 64 with no OSAS. OSAS was confirmed by polysomnography. Quantitative ultrasound (QUS) of calcaneum measuring speed of sound (SoS) and broadband ultrasound attenuation (BUA) were collected. Other parameters collected including bone profile, vitamin D levels, physical activity scoring and dietary calcium intake. Majority were male and Malay ethnicity. OSAS children were mostly obese (84%) and 57% had moderate to severe OSAS. Most had lower physical activities scores. Mean (SD) phosphate and Alkaline phosphatase were lower in OSA children compared to controls: PO_4_, *p* = 0.039 and ALP, *p* < 0.001. Using both single and multivariate analysis, children with OSAS had a lower mean SoS value, *p* < 0.001 and *p* = 0.004 respectively after adjusting for age, BMI and bone profile. Children with OSAS had lower SoS suggesting risk for secondary osteoporosis. QUS calcaneus is a non-invasive, feasible tool and can be used to screen risk of osteoporosis in children. Further bone mineral density assessment is needed in these groups of children to confirm diagnosis of osteoporosis.

## Introduction

Obstructive sleep apnoea syndrome (OSAS) is the most common form of sleep disordered breathing affecting 2–4% children^1^. The repetitive upper airway collapse with apnoea/hypopnea and recurrent hypoxia during sleep^2^ may cause multisystemic complications involving cardiovascular, endocrine, respiratory and neurocognitive dysfunction.

Secondary osteoporosis is a result of impaired bone mass, strength, and microarchitecture. Recent studies suggest that OSAS is a contributing factor for osteoporosis as hypoxia blocks the growth of osteoblasts and strongly stimulates osteoclasts resulting in thinning of bone that eventually becomes osteoporosis^3^. The repetitive hypoxia and subsequent reoxygenation phenomena seen in OSAS may also induce oxidative stress leading to acidosis due to reduced vascular perfusion^4^.

Dual-energy X-ray absorptiometry (DXA) scan assesses BMD which is gold standard to diagnose osteoporosis in children and adolescents^5,6^. However, in paediatric patients, the correct reading of DXA is affected by several limitations. The T-score parameter is not applicable to children because it refers to the BMD value upon peak bone mass (PBM) achievement. The z-score was adjusted for age, gender, and ethnic group, but it does not consider weight and height variability^7^. As a promising alternative to DXA, quantitative ultrasound (QUS) has recently emerged for evaluating bone health^8^. It is radiation-free, fast to execute and cheap. Through measurements of the speed of sound, broadband ultrasound attenuation, stiffness factor, and bone transmission time, QUS can give information not only on density but also bone elasticity, microarchitecture, and thickness^9,10^. An adult study done in Taiwan by Chen YL et al., demonstrated that people suffering from OSAS were 2.74 more times likely to get osteoporosis^11,12^. Another cross-sectional study performed by Uzkeser et al.^13^, investigating bone mineral density (BMD) in 21 male patients with OSAS and 26 control subjects, found a relationship between OSAS and osteoporosis. Although multiple adult studies have seen associations between these two diseases, the long-term implications of OSAS during early life and adolescence to the skeletal system has not been studied. Osteoporosis and fractures in children can lead to significant morbidity and reduce quality of life. Therefore, the aim of this study was to look at the risk for secondary osteoporosis in children with OSAS.

### Material and methods

We conducted a cross-sectional study using two-study groups aged 10–17 years: a group of newly diagnosed OSAS and a matched group of non-OSAS (control) between July 2016 to 2018. Children with OSAS were recruited from the Paediatric Sleep Centre at University Malaya Medical Centre (UMMC), Kuala Lumpur, Malaysia. The non-OSAS group comprised subjects that did not have any symptoms of sleep disordered breathing recruited from both  inpatient and outpatient paediatric patients who were admitted and attended clinics at UMMC. They were matched with the OSAS individuals in terms of age and gender.

Exclusion criteria for OSAS patients and controls were: children with dysmorphism; on continuous pressure airway pressure therapy; use of chronic corticosteroids over 4 weeks; immunosuppressant, anti-epileptics, chronic lung disease, chronic liver disease or chronic renal failure; concomitant neurological disease; type 1 diabetes mellitus, anorexia nervosa symptoms, thalassemia, sickle cell disease or signs of acute or chronic inflammatory disorders; calcium or vitamin D supplements; and a history of immobilization or fractures. This study was conducted in accordance with the guidelines and regulations by the UMMC Medical Ethics Research Committee (MREC ID: 20164-2371). All patients recruited had parent’s agreement and informed consent. We calculated that the sample size in each treatment group should be 36 at an alpha of 0.05 and a power of 0.80.

### Polysomnography

OSAS group were confirmed by overnight polysomnography (PSG) conducted according to American Academy of Sleep Medicine (AASM). A sleep study determines the rapid eye movement (REM) and non-REM sleep stages via the electroencephalogram and electro-oculogram signals; respiratory obstructive and central events from the airflow and thermistor cannulas, and inductance plethysmography; adequate gas exchange (oxygen saturation and carbon dioxide measurements).

The PSG data were analysed manually by a respiratory physiologist using computer software (Cadwell, USA). Recorded respiratory data included counts and indices of the following events: obstructive apnoea, obstructive hypopneas, central apnoea, and mixed apnoea during sleep. All respiratory events were also scored according to the respiratory rules for children in the AASM guidelines where an apnoea was scored when peak signal excursions drop by ≥ 90% of pre-event baseline using an oronasal thermal sensor (diagnostic study) and obstructive hypopnea was scored when there was a reduction in the signal excursions by ≥ 30%, desaturation by 3% or presence of an arousals^14^. The obstructive apnoea hypopnea index (OAHI) was defined as the number of obstructive apnoea, hypopneas, and mixed apnoea per hour. Children below 12-years were scored according to the paediatric OSA severity scoring criteria: mild OSA = OAHI ≥ 1.5 to < 5/h; moderate OSA = OAHI ≥ 5 to < 10/h; and severe OSA = OAHI ≥ 10/h and those >12 years were scored according to the adult criteria: mild OSA = OAHI ≥ 5 to < 15/h, moderate OSA= OAHI ≥ 15 to 30/h and severe OSA= OAHI ≥ 30/h. The central apnoea index (CAHI) was defined as the number of central apnoea and hypopneas per hour and a CAHI < 5 events/hour was considered normal^14^.

### Anthropometry

Body mass index (BMI) was calculated from measured height and weight. According to BMI, 18–24.9 kg/m ^2^ was accepted as normal weight, 25–29.9 kg/m ^2^ as overweight, 30–39.9 kg/m ^2^ as obese, and over 40 kg/m ^2^ as morbidly obese.

### Laboratory

All children had their serum bone profile (calcium, phosphate and alkaline phosphatase) measured. Normal values were 2.2 to 2.5 mmol/L for Ca, 1.0 to 1.75 mmol/L for Phosphate and 74 to 390 u/L for Alkaline Phosphatase. Vitamin D levels were measured as total 25 (OH)D nmol/L. We used the American Academy of Paediatric classification of the vitamin D level to define serum 25(OH)D status^15^; 25(OH)D < 50 nmol/L equates to Vitamin D Deficiency (VDD), while < 75 nmol/L equates to vitamin D insufficiency (VDI) and > 75 nmol/L denotes vitamin D sufficiency (VDS).

### Food frequency questionnaire (FFQ)

Subjects were asked to report their dietary intake over the previous 4 weeks^16^. The selected nutrients (expressed in mg) and total energy were estimated from the food composition table. According to the Food and Agriculture Organization of the United Nations & World Health Organization (FAO/WHO) (2002), the recommended nutrient intakes (RNI) for calcium in children between 10 to 18 years is set at 1000 mg/day. In addition, a non-quantitative FFQ was used to assess daily, weekly and monthly intake frequency of 109 commonly consumed foods. The FFQ was used to calculate the estimated calcium intake.

### Physical activity questionnaire for children/adolescent (PAQ-C/PAQ-A)

In order to measure and assess levels of physical activities, we applied the widely used questionnaire for children, PAQ-C^17^. The physical activity 7-day recall questionnaire was filled by the parents and subjects. For each question, the test scored of 1 indicates low physical activity and a value of 5 indicates high physical activity. The final PAQ-C activity score of the test is calculated as the mean value of the answers.

### Ultrasound calcaneum

We used a gel coupled calcaneal QUS device (GE Achilles EXPII) measuring the speed of sound (SOS) in m/s and broadband ultrasound attenuation (BUA) in dB/MHz. Performing a quality control using the protocol provided by the manufacturer ensured the stability of QUS parameters. We used the same calcaneal QUS device throughout the study with the same operator conducting measurements who was blinded to the PSG. It used high frequency sound waves (ultrasound) to evaluate bone status in the heel, the os calcis. Measurements were performed with the participants seated, with one foot placed on the Footplate. The heel was surrounded by warm water encapsulated between inflated membranes. A transducer on one side of the heel converts an electrical signal into a sound wave, which passes through the water and the person’s heel. A transducer at a fixed distance on the opposite side of the heel receives the sound wave and converts it to an electrical signal that was analysed. The SOS and BUA were then read off the densitometer screen and recorded. Slower speed of sound indicates longer transit time and bone is more likely to be osteoporotic.

### Statistical analysis

All statistical analyses were performed using SPSS version 23.0 (IBM Corp, New York, USA). In the descriptive analysis, continuous data of socio-demographic, anthropometry and nutrition information were represented in mean (standard deviation) or median (interquartile range) depending on the normality of the data where in all categorical data were expressed in percentage. Single factor multivariate analysis (MANOVA) was used to investigate the factor association between the categorical variables with the two-outcome variables namely broadband ultrasound attenuation (BUA) and speed of sound (SoS) measured from the quantitative ultrasound calcaneus. We used Pearson or Spearman correlation (depending on the normality of the data) to compare the study subject and matched control differences continuous variables with both outcome variables (BUA and SoS). Variance inflation factor analysis (VIF) was conducted to rule out multicollinearity issues among all factors and variables that were significantly correlated with the outcome variables. In addition, a Mahalanobis distance diagnosis tool was used to eliminate all outliers from this study. Lastly, a multivariate analysis of covariance (MANCOVA) was used to identify the true factors associated with BUA and SOS, respectively. A two-sided value of  *p* < 0.05 were considered significant.

### Ethics declarations

This study was approved by the UMMC Medical Ethics Research Committee (MREC ID: 20164-2371). All patients recruited had parent’s agreement and consent.

## Results

180 participants were initially identified however 30 were excluded as 20 did not meet the inclusion criteria and 10 participants had incomplete data. A total of 150 participants were analysed of which 86 had obstructive sleep apnoea syndrome (OSAS) and 64 were age and gender matched control group. The baseline demographic data and clinical characteristics of these patients are listed in Table 1. Mean ages of both groups were similar 12 ± 2 years. Majority of the patients were male (72%) and Malay (68%). The only significant difference seen between the two groups were the body mass index (*p* < 0.001). Children from OSAS group were mostly obese (88%) as compared to the control group (25%), *p* < 0.001. Of the 86 children with OSAS, 22 (25.6%) had mild OSAS, 27 (31.4%) had moderate OSAS and 37 (43%) had severe OSAS.

**Table 1:** Demographic and baseline characteristics for control and OSA groups (n = 150).

Variables	Control (n = 64)	OSA (n = 86)	*p*
**Age (mean, SD)**	12.98 (2.119)	12.90 (2.052)	0.796*
**Gender**			
Female	23 (35.9)	19 (22.1)	0.062^#^
Male	41 (64.1)	67 (77.9)	
**Ethnicity**			
Chinese	6 (9.4)	7 (8.1)	0.664^#^
Indian	17 (26.5)	18 (20.9)	
Malay	41 (64.1)	61 (70.9)	
**BMI**			
Underweight	6 (9.4)		< 0.001^#^
Normal	42 (65.6)	10 (11.6)	
Overweight/obese	16 (25)	76 (88.4)	
Vitamin D (nmol/ml)	50.03 (12.20)	46.59 (9.75)	0.066*
Serum calcium (mg/dL)	2.38 (0.13)	2.38 (0.15)	0.860*
Serum phosphate (mmol/L)	1.50 (0.18)	1.56 (0.16)	0.039*
Serum alkaline phosphatase (µ/L)	216.78 (58.59)	176.84 (57.31)	< 0.001*
**Physical activity (PAQ-C/A)**			
Low (score 1)	39 (60.9)	64 (74.4)	0.078^#^
High (score 5)	25 (39.1)	22 (25.6)	
Calcium intake (mg/day) (FFQ), mean (SD)	1029.06 (117.98)	1029.88 (149.19)	0.971*

All continuous variables skewness and kurtosis were in the range between − 2 and 2 hence the data were represented by mean (SD) where *SD* standard deviation, *t* , student t-test*; x^2^ , chi square^#^; *p* p value.

Both groups had mostly lower level physical activities (Table 1), with physical activity scoring, score 1 (PAQ-C). There was no statistical difference in physical activities between children with and without OSAS (*p* = 0.078). The mean serum calcium of both groups was within normal limits 2.38 mg/dL. Vitamin D levels were low in both groups but lower in the OSAS group (*p* = 0.06). Alkaline phosphatase although within the normal limits, was significantly reduced in the OSA group, *p* < 0.001.

Ultrasound calcaneum measurements showed OSA group to have a significant slower SoS as compared to control however there were no difference in BUA (Table 2). Table 3 shows the comparison of the parameters of QUS of the calcaneus [speed of sound (SoS) and broadband ultrasound attenuation (BUA)] in children with difference severity of OSAS (n = 86). There were no statistical difference seen in the severity with this two parameters. Single factor multivariate analysis (MANOVA) revealed that factors such as BMI (Wilk’s lamba = 0.866, F_(2,147)_ = 11.405, *p* =  < 0.001) and OSA (Wilk’s lamba = 0.863, F_(2,147)_ = 11.698, *p* =  < 0.001) were significantly associated with the combination of BUA and SoS (Table 4).

**Table 2:** Comparison of the parameters of QUS of the calcaneus (speed of sound (SoS) and broadband ultrasound attenuation (BUA) in children with OSAS to healthy control.

	OSAS (n = 86)	Control (n = 64)	*p* value
SoS (m/sec), mean (SD)	1538.71 ± 25.79	1558.34 ± 28.70	< 0.001
BUA db/MHz, mean (SD)	112.71 ± 14.14	109.758 ± 16.48	0.24

Remarks : independent t-test was used to investigate the mean differences for BUA and SOS (normal distribution based on skewness and kurtosis in between − 2 and 2). Equal variances were assumed (*p* > 0.05).

*SD* standard deviation.

**Table 3:** Comparison of the parameters of QUS of the calcaneus [speed of sound (SoS) and broadband ultrasound attenuation (BUA) in children with difference severity of OSAS (n = 86)].

	Mild OSA (n = 22)	Moderate OSA (n = 27)	Severe OSA (n = 37)	*p* value
SoS (m/s), mean (SD)	1533.05 (28.09)	1533.36 (21.47)	1545.97 (26.10)	0.075
BUA db/MHz, mean (SD)	113.90 (12.67)	115.32 (16.23)	110.10 (13.25)	0.314

Remarks : one-way ANOVA was used to investigate the mean differences for BUA and SOS (normal distribution based on skewness and kurtosis in between − 2 and 2) among children with difference severity of OSAS. Equal variances were assumed (*p* > 0.05).

*SD* standard deviation.

**Table 4:** Comparison of quantitative ultrasound (BUA and SoS) with demographic factors and OSA (n = 150) by single factor multivariate analysis (MANOVA).

Factor	Box’s M (*p* value)	Levene’s test F_(1,148)_ (*p* value)	Wilks’s lambda	F_(2,147)_	*p* value
**OSA**					
Control (n = 64)	2.836 (0.425)	BUA : 2.215 (0.147)	0.863	11.698	< 0.001
OSAS (n = 86)		SOS : 0.414 (0.521)			
**Body mass index (BMI)**					
Normal/thin (n = 58)	1.401 (0.710)	BUA : 0.219 (0.640)	0.866	11.405	< 0.001
Overweight/obese (n = 92)		SOS : 0.604 (0.438)			
**Gender**					
Female (n = 42)	2.333 (0.515)	BUA : 2.759 (0.099)	0.985	1.082	0.341
Male (n = 108)		SOS : 0.336 (0.563)			
**Ethnicity**					
Malay (n = 102)	15.039 (0.002)	BUA : 0.296 (0.587)	0.987	0.964	0.384
Non Malay (n = 48)		SOS : 0.117 (0.733)			

*Box’s M*  Box’s test for equivalence of covariance matrices, *F* F statistic.

Pearson correlation identified continuous variables like age (r = 0.245, *p* =  < 0.01) and BMI (r = 0.205, *p* < 0.05) had a positive relationship with the increase of BUA however BUA was negatively correlated with the vitamin D level (r = − 0.165, *p* =  < 0.05) and also the alkaline phosphatase level (− 0.162, *p* =  < 0.05). Meanwhile, other outcome variable (SoS) had a positive correlation with age (r = 0.208, *p* =  < 0.05) and had a negative relationship with BMI (r = − 0.248, *p* =  < 0.01).

Table 5 showed the multiple variables multivariate covariance analysis on BUA and SoS. Overall, the combined dependent variables of BUA and SOS were different among participants with OSAS compared with those in control group (Wilk’s Lambda = 0.940; F(2,143) = 4.526; *p* = 0.012) after adjusted by other factors like age, BMI, vitamin D level and alkaline phosphatase level. Note that the main effect of OSA on BUA was not significant [F(1,144) = 1.547; *p* = 0.216] but was significantly associated with SoS [F(1,144) = 8.424; *p* = 0.004] after controlled by the other variables. The total variances explained by the model corresponding to BUA and SOS were adjusted R^2^ = 10.7% and adjusted R^2^ = 15.3%, respectively.

**Table 5:** Multiple variables multivariate covariance analysis (MANCOVA) on BUA and SoS (n = 150).

Main factors	BUA		SOS	
	Adjusted mean (SE)	Adjusted mean difference (lower CI, upper CI)	Adjusted mean (SE)	Adjusted mean difference (lower CI, upper CI)
Control	113.83 (2.24)	4.15 (− 2.45, 10.75)	1557.28 (4.12)	17.79 (5.67, 29.90)**
OSA	109.68 (1.85)		1539.50 (3.39)	

Other factors	Adjusted B (SE)	Adjusted B (lower CI, upper CI)	Adjusted B (SE)	Adjusted B (lower CI, upper CI)
Age	1.72 (0.59)	1.72 (0.55, 2.90) **	2.83 (1.09)	2.83 (0.67, 4.98) *
BMI	2.99 (1.11)	2.99 (0.81, 5.17) **	− 0.72 (2.03)	− 0.72 (− 4.73, 3.29)
Vitamin D level	− 0.15 (0.11)	− 0.15 (− 0.37, 0.07)	− 0.39 (0.20)	− 0.39 (− 0.78, 0.01)
Alkaline phosphatase	− 0.02 (0.02)	− 0.02 (− 0.07, 0.02)	0.04 (0.04)	0.04 (− 0.04, 0.12)
Dietary calcium intake	2.39 (1.01)	2.39 (0.88, 4.55)	1.55 (1.45)	1.55 (0.35, 2.68)
Physical activity	1.33 (0.68)	1.33 (0.45, 3.45)	2.35 (1.23)	2.35 (0.65, 3.45)

Adjusted R^2^ for BUA = 0.107; adjusted R^2^ = for SOS = 0.153.

*SE* standard error, *B* regression coefficient, *CI* confidence interval.

*p < 0.05.

**p < 0.01.

## Discussion

Many studies have reported OSAS to be comorbid with cardiovascular disease, hypertension, type 2 diabetes mellitus, and insulin resistance, but few have investigated the possible relationship between OSAS and bone disease, especially in children. Studies evaluating the incidence or prevalence of osteoporosis in OSAS were mostly performed in adult but limited in children. This study is the first to evaluate association of OSAS in children and its risk of secondary osteoporosis.

Hypoxia, a characteristic of OSAS syndrome results in changes in bone turnover, and recent in vitro studies have shown that hypoxia promotes osteoclast formation and activity and inhibits osteoblast function, thus determining bone resorption^18,19^. Thus, this process occurring during an individual's growth years can alter bone development, mineralization leading to insufficient peak bone mass (PBM)^20^ resulting in osteopenia/osteoporosis. Tomiyama et al.found that bone resorption markers were significantly higher in their OSAS patients compared with their control subjects and these markers reversed after 3 months of continuous positive airway pressure therapy^21^.

Our study, the first in children, showed children with OSAS had a slower speed of sound (SoS) compared to the control group after adjusting for age, BMI and bone profile. However, there were no statistical differences seen with BUA in both groups. There were also no association between both SoS and BUA with other factors such as physical activity, ethnicity, gender and calcium intake. According to the International Society of Clinical Densitometry (ISCD), calcaneal QUS is the only recognized measurement of QUS as the determinant of bone health status^19^. Precision in QUS variables measured in children is reported to be better for SoS than BUA^13, 21,22^. Toyras et al. examined the relationship between calcaneal QUS and bone properties and found that SoS was closely related to BMD and had significant correlations with microarchitecture indices of the bone^8,23,24^. A lower speed of sound suggests a lower bone density and other factors that can cause early osteoporosis needs to be identified and manage to avoid future complications. There was no correlation seen between the severity of OSAS with SoS and BUA.

In our current study, majority of the participants demonstrated low physical activity score. Since OSAS patients and control population did not differ in terms of physical activity and age we can exclude insufficient physical activity as putative factors of reduced BMD in OSAS patients. We also showed there were no obvious differences in the bone profile between the two groups except serum alkaline phosphatase (ALP). The mean serum alkaline phosphatase in the OSA group was significantly lower indicating low bone matrix and bone formation. However, ALP as a single marker is non-specific and needs to be correlated with other specific parameters to identify the exact pathology of the bone.

This study also demonstrated high prevalence of vitamin D deficiency in both groups, with the OSAS group having a lower level^25^. Two local studies, SEANUTS and Majid et al. also demonstrated high prevalence of vitamin D insufficiency and deficiency in Malaysian children^26^. This is rather interesting being a tropical country. One possible explanation for this could be due to a more sedentary lifestyle resulting in increase in weight gain and reduced sun exposure. Therefore, supplementation of vitamin D would need to be considered in this group of children considering high prevalence of Vitamin D deficiency.

This study has a few limitations. In the OSAS group, majorities were obese compared to the control group which could explain the low Vitamin D levels^25^. Obesity increases the risk for OSAS which in may turn worsen obesity because of sleep and metabolic derangements^27–29^. Obesity is also associated with low grade chronic inflammation^30–32^ and the increased cytokines such as TNF- ∝ , IL-6, and IL-1 promotes osteoclast activity and bone resorption. Although the results of studies of obesity and bone metabolism are inconsistent, we considered obesity to be a possible confounding factor. We did not look at pubertal status of our patients which is also another study limitation. The greatest accretion of bone occurs during puberty; therefore, any deficiencies may contribute towards osteoporosis. In our study, the patient exclusion criteria would have mitigated this issue. The patient convenience sampling performed in this study is rather bias as the OSA patients were recruited from the sleep laboratory who are likely to be obese with ongoing systemic inflammation increasing the risk of low bone mineral density. The absence of an interventional treatment, such as positive airway pressure (PAP), is another limitation of this study since we cannot test the possible restorative effect of PAP therapy on BMD in OSA patients.

## Conclusion

This study suggests that children with OSAS have lower speed of sound and may be at risk of secondary osteoporosis. Identifying children at risk of secondary osteoporosis is warranted as fractures can lead to significant morbidity and reduced quality of life. As more and more children are diagnosed with obstructive sleep apnoea worldwide, more attention should be focused on the relationship between obstructive sleep apnoea and bone health so we can identify those at risk and prevent osteoporosis.

## Data Availability

No datasets were generated or analysed during the current study.

## References

[1] Chang SJ, Chae KY (2010). Obstructive sleep apnea syndrome in children: Epidemiology, pathophysiology, diagnosis and sequelae. Korean J. Pediatr..

[2] Labarca G, Cruz NR, Descalzi F (2014). Multisystemic involvement in obstructive sleep apnea. Rev. Med. Chil..

[3] Ward LM, Konji VN, Ma J (2016). The management of osteoporosis in children. Osteoporos. Int..

[4] Suzuki YJ, Jain V, Park AM, Day RM (2006). Oxidative stress and oxidant signaling in obstructive sleep apnea and associated cardiovascular diseases. Free Radic. Biol. Med..

[5] Bianchi ML, Baim S, Bishop NJ, Gordon CM, Hans DB, Langman CB (2010). Official positions of the International Society for Clinical Densitometry (ISCD) on DXA evaluation in children and adolescents. Pediatr. Nephrol..

[6] Wasserman H, O'Donnell JM, Gordon CM (2017). Use of dual energy X-ray absorptiometry in pediatric patients. Bone.

[7] Zemel BS, Kalkwarf HJ, Gilsanz V, Lappe JM, Oberfield S, Shepherd JA (2011). Revised reference curves for bone mineral content and areal bone mineral density according to age and sex for black and non-black children: Results of the bone mineral density in childhood study. J. Clin. Endocrinol. Metab..

[8] Teitelbaum SL (2000). Bone resorption by osteoclasts. Science.

[9] Wang KC, Wang KC, Amirabadi A, Cheung E, Uleryk E, Moineddin R (2014). Evidence-based outcomes on diagnostic accuracy of quantitative ultrasound for assessment of pediatric osteoporosis—A systematic review. Pediatr. Radiol..

[10] Weeks BK, Hirsch R, Nogueira RC, Beck BR (2016). Is calcaneal broadband ultrasound attenuation a valid index of dual-energy x-ray absorptiometry-derived bone mass in children?. Bone Jt. Res..

[11] Wang TY, Lo YL, Chou PC, Chung FT, Lin SM, Lin TY (2015). Associated bone mineral density and obstructive sleep apnea in chronic obstructive pulmonary disease. Int. J. Chron. Obstruct. Pulmon. Dis..

[12] Chen YL, Weng SF, Shen YC, Chou CW, Yang CY, Wang JJ (2014). Obstructive sleep apnea and risk of osteoporosis: A population-based cohort study in Taiwan. J. Clin. Endocrinol. Metab..

[13] Uzkeser H, Yildirim K, Aktan B, Karatay S, Kaynar H, Araz O (2013). Bone mineral density in patients with obstructive sleep apnea syndrome. Sleep Breath.

[14] Berry RB, Budhiraja R, Gottlieb DJ, Gozal D, Iber C, Kapur VK (2012). Rules for scoring respiratory events in sleep: Update of the 2007 AASM manual for the scoring of sleep and associated events. Deliberations of the sleep apnea definitions Task Force of the American Academy of Sleep Medicine. J. Clin. Sleep Med.

[15] Lee JY, So TY, Thackray J (2013). A review on vitamin d deficiency treatment in pediatric patients. J. Pediatr. Pharmacol. Ther..

[16] Weir RR, Carson EL, Mulhern MS, Laird E, Healy M, Pourshahidi LK (2016). Validation of a food frequency questionnaire to determine vitamin D intakes using the method of triads. J. Hum. Nutr. Diet.

[17] Janz KF, Lutuchy EM, Wenthe P, Levy SM (2008). Measuring activity in children and adolescents using self-report: PAQ-C and PAQ-A. Med. Sci. Sports Exerc..

[18] Utting JC (2006). Hypoxia inhibits the growth, differentiation and bone-forming capacity of rat osteoblasts. Exp. Cell Res..

[19] Arnett TR (2010). Acidosis, hypoxia and bone. Arch. Biochem. Biophys..

[20] Bachrach LK (2001). Acquisition of optimal bone mass in childhood and adolescence. Trends Endocrinol. Metab..

[21] Tomiyama H, Okazaki R, Inoue D, Ochiai H, Shiina K, Takata Y (2008). Link between obstructive sleep apnea and increased bone resorption in men. Osteoporos. Int..

[22] Parfitt AM (2002). Misconceptions (2): Turnover is always higher in cancellous than in cortical bone. Bone.

[23] Laugier L, Wear KA, Waters KR (2008). Introduction to the special issue on diagnostic and therapeutic applications of ultrasound in bone—Part I. IEEE Trans. Ultrason. Ferroelectr. Freq. Control.

[24] Bates DW, Black DM, Cummings SR (2002). Clinical use of bone densitometry: Clinical applications. JAMA.

[25] Kheirandish-Gozal L, Peris E, Gozal D (2014). Vitamin D levels and obstructive sleep apnoea in children. Sleep Med..

[26] Poh BK (2013). Nutritional status and dietary intakes of children aged 6 months to 12 years: Findings of the Nutrition Survey of Malaysian Children (SEANUTS Malaysia). Br. J. Nutr..

[27] Al-Sadat, N. *et al*. Vitamin D deficiency in Malaysian adolescents aged 13 years: Findings from the Malaysian Health and Adolescents Longitudinal Research Team study (MyHeARTs). *BMJ Open* **6**(8), e010689-2015-010689 (2016). 10.1136/bmjopen-2015-010689 PMC5013370 27540095

[28] Newman AB (2005). Progression and regression of sleep-disordered breathing with changes in weight: The Sleep Heart Health Study. Arch. Intern. Med..

[29] Foster GD (2009). Obstructive sleep apnea among obese patients with type 2 diabetes. Diabetes Care.

[30] Romero-Corral A, Caples SM, Lopez-Jimenez F, Somers VK (2010). Interactions between obesity and obstructive sleep apnea: Implications for treatment. Chest.

[31] Mariani S (2012). Obstructive sleep apnea and bone mineral density in obese patients. Diabetes Metab. Syndr. Obes..

[32] Zamarron C, Garcia Paz V, Riveiro A (2008). Obstructive sleep apnea syndrome is a systemic disease. Current evidence. Eur. J. Intern. Med..

